# Efficacy of different interaction devices using non-immersive virtual tasks in individuals with Amyotrophic Lateral Sclerosis: a cross-sectional randomized trial

**DOI:** 10.1186/s12883-018-1212-3

**Published:** 2018-12-17

**Authors:** Isabela Lopes Trevizan, Talita Dias Silva, Helen Dawes, Thais Massetti, Tânia Brusque Crocetta, Francis Meire Favero, Acary Souza Bulle Oliveira, Luciano Vieira de Araújo, Ana Carolina Costa Santos, Luiz Carlos de Abreu, Shelly Coe, Carlos Bandeira de Mello Monteiro

**Affiliations:** 1 Department of Speech Therapy, Physiotherapy and Occupational Therapy – Faculty of Medicine, University of São Paulo, Rua Cipotânea, 51, São Paulo, CEP: 05360-000 Brazil; 2 Federal University of São Paulo – Paulista School of Medicine, Rua Sena Madureira, 1500, São Paulo, CEP: 04021-001 Brazil; 3 Institute of Nursing and Allied Health Research, Oxford Brookes University, Headington Campus, Oxford, OX3 0BP UK; 4 Department of Clinical Neurology, University of Oxford, Oxford, OX3 9DU UK; 5 Department of Physiotherapy, Speech Therapy and Occupational Therapy – Faculty of Medicine, University of São Paulo, Rua Cipotânea, 51, São Paulo, CEP: 05360-000 Brazil; 6 Department of Scientific Writing, Faculty of Medicine ABC, Avenida Príncipe de Gales, 821, Santo André, São Paulo, CEP: 09060-650 Brazil; 7 School of Arts, Sciences and Humanities, University of São Paulo, Rua Arlindo Bettio, 1000, São Paulo, CEP: 038-28-000 Brazil

**Keywords:** Amyotrophic lateral sclerosis, Virtual reality exposure therapy, User-computer Interface, Rehabilitation, Motor activity

## Abstract

**Background:**

Amyotrophic Lateral Sclerosis (ALS) is a rapid progressive neurodegenerative disease, characterized by a selective loss of motor neurons, brain stem and spinal cord which leads to deterioration of motor abilities. Devices that promote interaction with tasks on computers can enhance performance and lead to greater independence and utilization of technology.

**Objective:**

To evaluate performance on a computer task in individuals with ALS using three different commonly used non-immersive devices.

**Method:**

Thirty individuals with ALS (18 men and 12 women, mean age 59 years, range 44–74 years) with a mean score of 26, (minimum score of 14 and maximum 41) on the Revised Amyotrophic Lateral Sclerosis Functional Rating Scale (ALSFRS-R) and 30 healthy controls matched for age and gender, participated. All participants were randomly divided into three groups, each using a different device system (motion tracking, finger motion control or touchscreen) to perform three task phases (acquisition, retention and transfer).

**Results:**

Both the ALS and control group (CG) showed better performance on the computer task when using the touchscreen device, but there was limited transfer of performance onto the task performed on the Finger Motion control or motion tracking. However, we found that using the motion tracking device led to transfer of performance to the touchscreen.

**Conclusion:**

This study presents novel and important findings when selecting interaction devices for individuals with ALS to access technology by demonstrating immediate performance benefits of using a touchscreen device, such as improvement of motor skills. There were possible transferable skills obtained when using virtual systems which may allow flexibility and enable individuals to maintain performance overtime.

**Trial registration:**

Registration name: Virtual Task in Amyotrophic Lateral Sclerosis; Registration number: NCT03113630; retrospectively registered on 04/13/2017. Date of enrolment of the first participant to the trial: 02/02/2016.

## Background

Amyotrophic Lateral Sclerosis (ALS) is a rapid progressive neurodegenerative disease, characterized by a selective loss of motor neurons, brain stem and spinal cord, with death usually occurring 2 to 5 years after the onset of symptoms [1]. Studies suggest a worldwide incidence of 2/100.000 individuals with ALS per year and prevalence of 3–8 cases per 100.000 inhabitants [2, 3]. The main symptoms of ALS are consequences of lesions involving multiple regions in the spinal cord and brainstem, and include weakness, spasticity, pathological reflexes, fasciculations, cramps, and muscle atrophy. Loss of respiratory muscle innervation and associated complications are the most frequent causes of death [4]. Individuals often develop weakness of the intrinsic muscles of the hand, affecting precise movements such as grasping and manipulating objects [5, 6]. Early deterioration of speech intelligibility and the described weakness of the hands and upper limbs can leave individuals with ALS with difficulty performing functional tasks affecting communication, leisure, work and social activities [7, 8]_._

There are different approaches to improving quality of life for individuals with ALS, the highly recommended is the use of assistive and augmented technology, but is still hard to address most part of the needs of patients with ALS [9]. Products used include commercial products and more bespoke technologies such as Virtual Reality (VR) [10], characterized as a computer technology that provides three-dimensional artificial sensory feedback, in which the user engages in experiences similar to real-life tasks [11]. This has been implemented to support individuals to gain improvements in performance. The classification of VR ranges from non-immersive to completely immersive depending on how isolated from the physical environment the individual is, when interacting with the virtual environment [11–14]. VR encourages movement in three dimensions of space and can be similar to movements that occur in the real world [15]. Several non-immersive videogame systems have been developed for use at home, making the use of this technology less expensive and more accessible for different rehabilitation interventions [14].

Because of these characteristics, non-immersive VR systems have been studied as a therapeutic tool for individuals with ALS and have shown potential to promote motor improvements even in advanced stages of the disease [16, 17]. Other approaches include using different hardware devices for interacting with virtual tasks, such as a standard computer mouse for writing in a computational activity [18], and eye tracking technology to facilitate social interaction [19]. The evidence highlights that these types of devices can be used to preserve mental autonomy, influence psychological well-being and may modify disease course and influence end-of-life-decisions in severely affected patients with ALS [20] and those with locked-in syndrome [21]. The type of interaction device is important as it may affect the ease of engagement and use of VR. Alternative products include a touch screen (that offers tactile feedback) or virtual devices that are not dependent on touch (sensors that capture body movements, with no tactile feedback), but require higher motor and cognitive demand to use. The latter feature may promote retention and transfer of the performance to tasks that require similar motor and cognitive abilities. Considering the motor difficulties present in individuals with ALS, the characteristics of the different devices allow individuals to communicate, socialize and maintain autonomy and independence [22]. VR systems allow opportunities to optimize interactions and participate in rehabilitation through the supervision of a professional to customize the difficulty and repetitions of the task and specify interaction according to the current and changing physical needs and abilities of the individual [23–25].

Despite the presence of existing work with interaction systems, we have not found research informing patients or clinicians as to the importance of using specific commercially available VR systems for supporting performance. In order to develop evidence to underpin clinical guidance and inform patients, we set out to investigate the use of the different interaction devices [26] that may improve performance of individuals with ALS. Thus, we used a VR computer task to explore three non-immersive recent technological devices available commercially that could enable functionality on daily life tasks for individuals with ALS: (1) Motion tracking device consisting of a motion sensor developed to allow players to interact with the electronic games without the need to have a control/joystick in hand; (2) Finger motion control device consisting of an infrared sensor and cameras to capture precise and simultaneous movements of the fingers within hundredths of a millimeter; (3) Touchscreen, found in different technological devices such as tablets, computers and cell phones, which allow user interaction but with the need of the participant to touch the device to execute a task.

We anticipated that using an interface which requires a wide range of movement such as motion tracking device and touchscreen would require greater motor demand and energetic expenditure to perform a computer task in individuals with ALS, promoting low performance compared with interfaces that require a small range of movement such as finger motion sensor systems. It is expected that this effect would not be observed to the same degree in healthy controls and that they will perform better on touchscreen, as they have no motor deficits and this kind of interface is widely used in daily life [27]. Thus, the aim was to compare touchscreen, motion sensor and finger motion sensor systems and to identify which low-cost device enabled better performance and functionality in both individuals with ALS and a healthy control group. The results will provide information for use of the best device to enable utilization and improve upper limb functional tasks, and inclusion in daily life activities of individuals with ALS.

## Method

### Participants

Between February and December of 2014, a total of 60 individuals participated (convenience sample), 30 individuals with ALS (18 men and 12 women, mean age 59 years, range 44–74 years) and 30 healthy individuals who formed the control group, which were (equally) matched individually for age and gender with the ALS group. The inclusion criteria of this study were individuals diagnosed with ALS defined according to the revised classification of *El Escorial* [28, 29] who regularly attended the Neuromuscular Disease Research Sector (SIDNM) of the Federal University of São Paulo. Considering that the device systems offer motion capture in three dimensions with high sensitivity to movement and touch, even individuals with limited movement and with worse scores on functional scales could perform the task and present improvement of performance, so we could not use functional scales scores as exclusion criteria. Thus, exclusion criteria were individuals not capable of performing the virtual task in a single trial-test (verbal and written instructions provided before the experiment), or motor contractures on upper limbs that prevented handling the devices chosen for the study. In order to have a more homogeneous group, we also excluded individuals with Bulbar-onset ALS, familial ALS, progressive muscular atrophy (PMA), primary lateral sclerosis (PLS) and locked-in syndrome.

### Rating scales

For clinical characteristics of the participants, three scales referring to functional assessment, fatigue and quality of life were applied:

1. As a functional assessment tool, the Revised Amyotrophic Lateral Sclerosis Functional Rating Scale (ALSFRS-R) [30] was used, validated in Brazilian individuals with ALS which allows monitoring of symptoms and limitations of daily living activities. The scale has 12 questions, with scores ranging between 0 and 4, and a maximum score of 48 (where the participant is in his or her best state) [19, 30].
2. To evaluate fatigue during the execution of tasks, we used the Fatigue Severity Scale (FSS). The FSS contains nine statements, and, for each item, participants are instructed to choose a score ranging from 1 to 7, 7 representing the highest level of agreement with a given statement. The total FSS score is obtained by calculating the mean of all items, a score ≥ 4 indicating the presence of fatigue [31].
3. For the assessment of quality of life, we used the Amyotrophic Lateral Sclerosis Assessment Questionnaire in the Portuguese Language (ALSAQ-40/BR), which is also validated for the Brazilian population with ALS [32]. The objective of this questionnaire is to assess health related quality of life in studies of patients with ALS. The instrument contains 40 questions that measure five areas of health state: 1 - Mobility (10 items), 2 - Daily Life Activities - ADLs (10 items), 3 - Feeding and Deglutition (3 items), 4 - Communication (7 items) and 5 - Emotional Aspects (10 items). Scale scores range from 0 to 100 within each domain. A score of 0 to 19 in a domain means that the patient presents no difficulty; 20 to 39, rarely presents difficulty; 40 to 59, sometimes presents difficulty; 60 to 79, often presents difficulty; and 80 to 100 always presents difficulty [33]. Thus, the questionnaire informs that a score closer to zero equates with better the quality of life, and the closer to one hundred, the more compromised the quality of life [32].

### Protocol

Individuals were randomly divided into three groups (simple randomization), using different interfaces for the acquisition of movement. All participants completed the study. The description of the VR task and the interfaces that were used are described below.

### Task

**Fig. 1:**
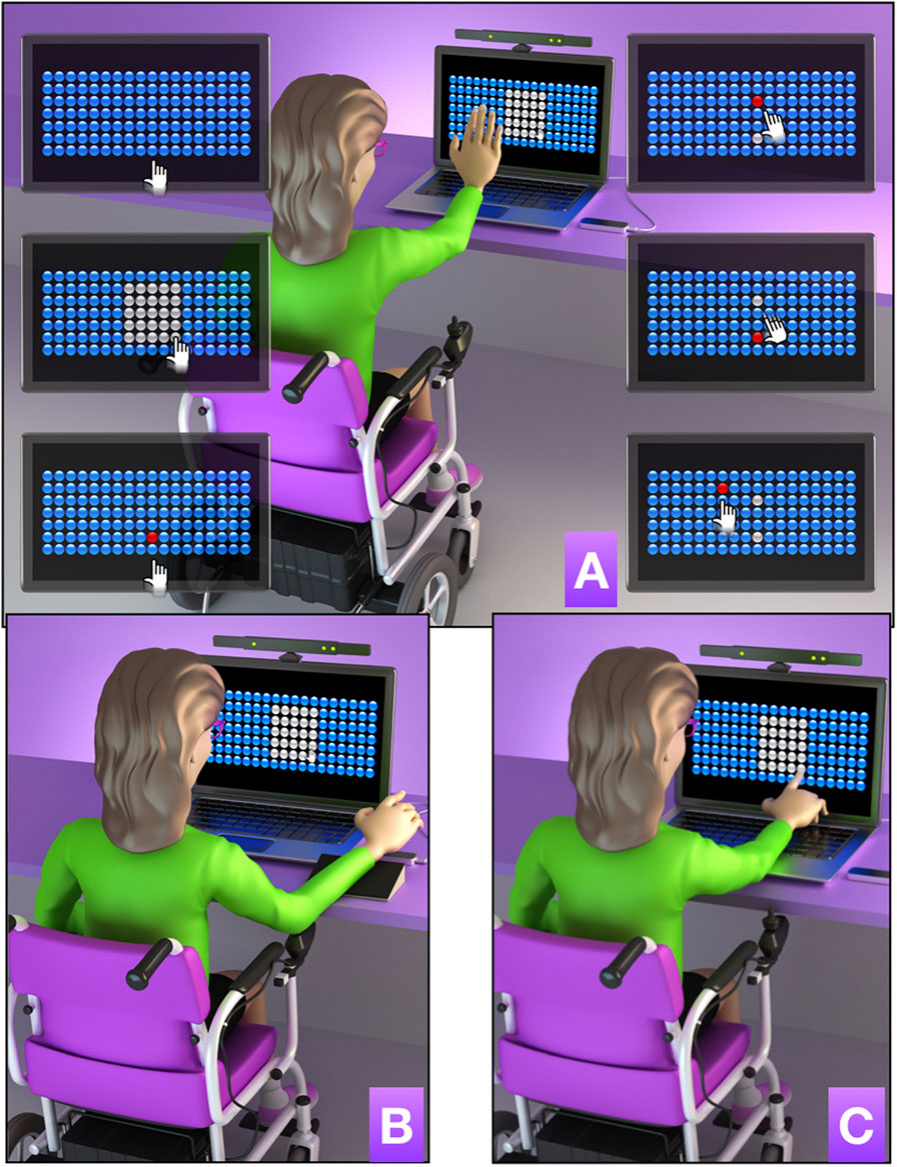
Graphic representation of an individual with ALS using the motion tracking (**a**), the finger motion control (**b**) and the touchscreen interface (**c**) during a proposed virtual task. a (upper left) initial screen of the task with 126 bubbles; (middle left) individual defines the skill zone by touching the screen for 10 s; (bottom left) researcher defines a target bubble in the center of bottom of the range line; (upper right) individual touches the bubble that appears randomly (in the chase area); (middle right) a return to bubble target; (bottom right) some touches are in a bubble outside the chase area, challenging the limits of the individual. The protocol was the same for all interfaces

**Fig. 2:**
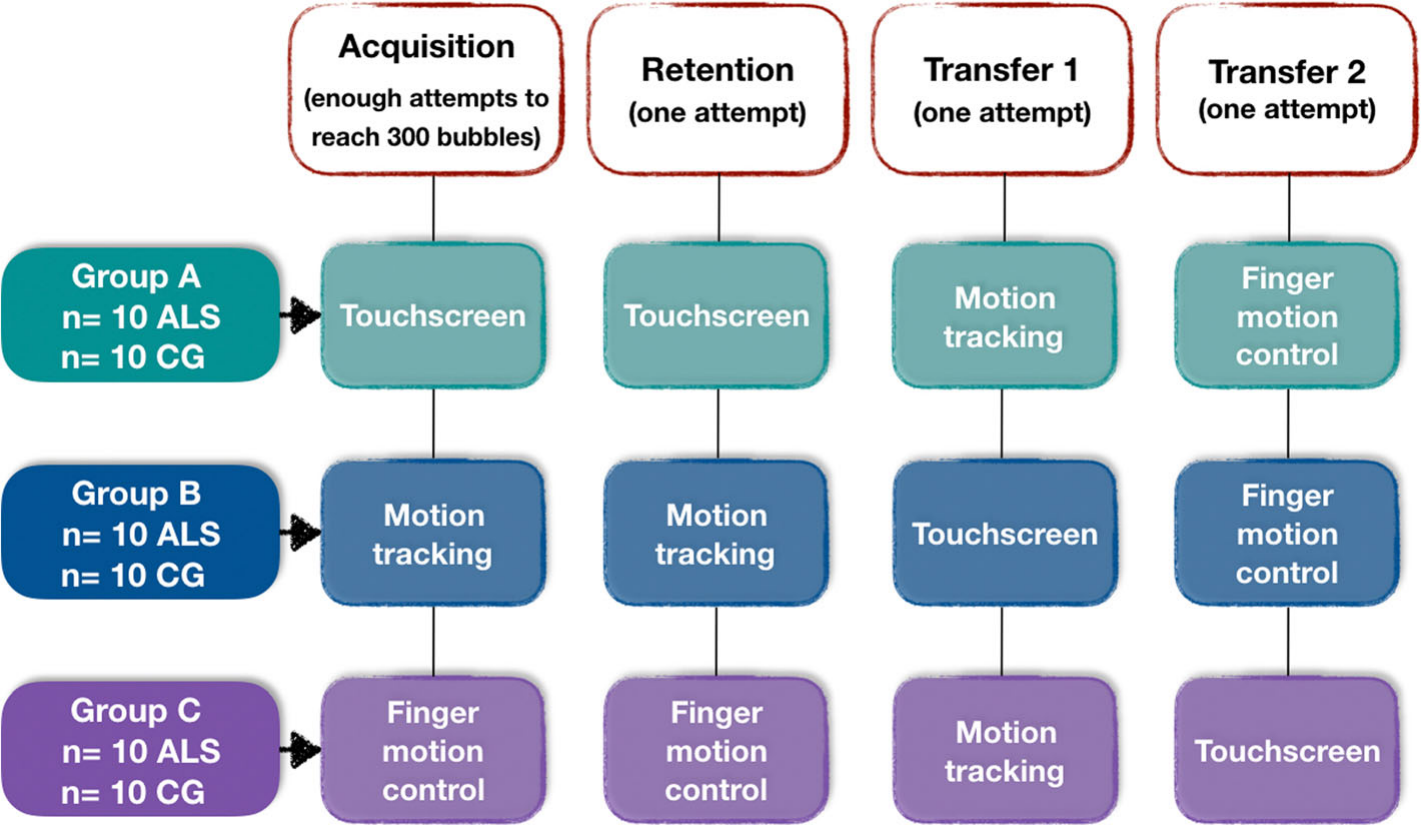
Study design. *ALS* Amyotrophic Lateral Sclerosis group, *CG* Control group

**Fig. 3:**
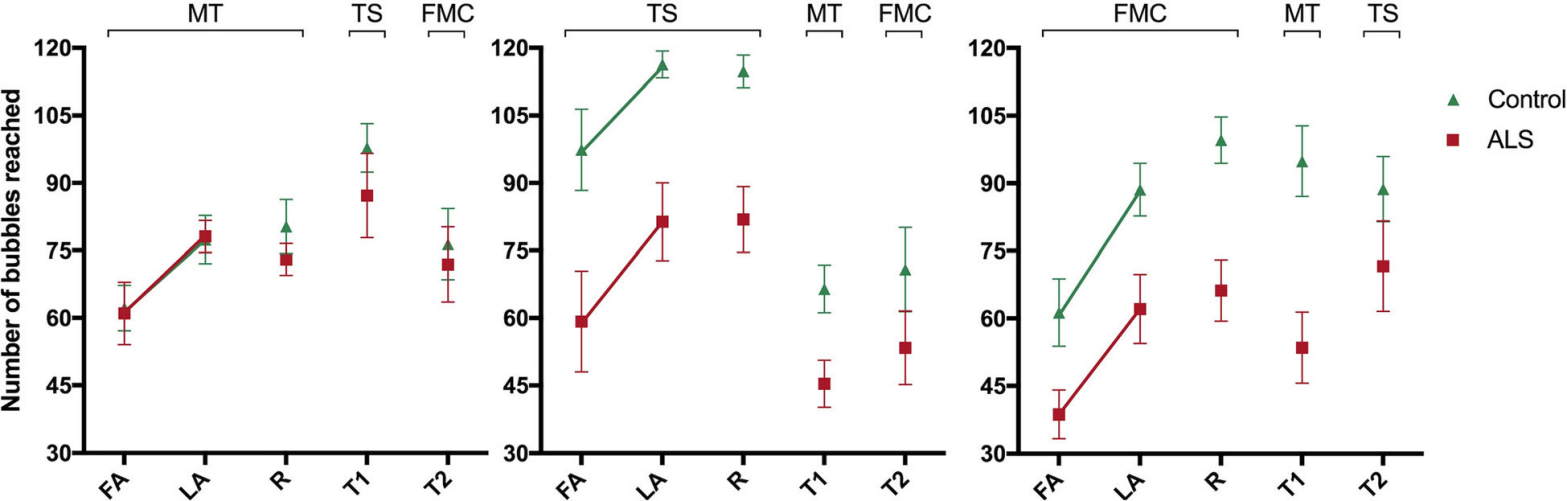
Representation (mean and standard error) of touched bubbles in all phases of the study in both groups: ALS and control. MT: Motion Tracking interface used; TS: Touchscreen interface used; FMC: Finger Motion Control interface used; FA: First attempt in the acquisition phase; LA: Last attempt in the acquisition phase; R: Tentative of retention phase; T1: First attempt at transfer phase with interface change; T2: Second attempt at transfer phase with the third interface; ALS: Amyotrophic Lateral Sclerosis group; Control: healthy control group

Participants performed a computer task to explore the potential of VR devices for enabling technology utilization developed by the Information Systems Team at the University of São Paulo. The task was set up as a game (with 3D images) in which the goal was to reach as many bubbles displayed on an 11-in. computer monitor, forming seven rows and 18 columns with a total of 126 bubbles (Fig. 1). These required individuals to be able to use a range as is typically required in VR systems. The task was divided into two phases: (1) the first phase was characterized by identification of dexterity zone or range of reach, in which the individuals had to touch (“burst”) the largest possible number of bubbles (changing bubble color from blue to gray) in a set time of 30 s, identifying the range zone (Figs. 1b, 2b and 3b); (2) the second phase was characterized as the persecution stage where the researcher defined a central bubble (usually chosen in the center of the skill area, on the bottom line), which changed the color to red (Figs. 1c, 2c and 3c). From this moment, the individual pursued random bubbles that appeared in their range zone (Figs. 1d, 2d and 3d), alternating with a return to the central bubble (Figs. 1e, 2e and 3e). This phase was carried out for 30 s. To challenge the participant, the task randomly provided bubbles outside the range zone (Figs. 1f, 2f and 3f) and generated a greater challenge to the individual. The software generated information of the coordinates x, y (row and column) including where the bubble was touched and the time the bubble was touched. During task execution, the participant received feedback on the number of bubbles touched, the remaining playing time and the total number of points obtained in attempts.

### Interfaces

Three different devices were coupled, and each group started the testing (acquisition phase) with a different device and then during the transfer phase used the other two devices. The devices were: Two non-contact devices, capable of capturing the movements performed by individuals in three dimensions called Motion tracking, (Fig. 1a). This composed of (1) a traditional Red, Green and Blue sensor (RGB) with the main purpose being for the sensing, representation and display of images in electronic systems such as a computer) and an infrared (IR) depth sensor that can measure the heat of an object as well as detecting motion (Kinect®, Microsoft) [34, 35] and (2) the finger motion control, (Fig. 1b), composed of infrared light-emitting diode (LED) characterized as a small motion sensor that demonstrated robustness to measure the movements of the hand and fingers (Leap Motion Control®) [36, 37].

(3) The touchscreen was the interface with physical contact on the computer screen itself, where individuals touched the screen to burst the bubbles (Fig. 1c). The touchscreen is a sensitive interface employed through pressure created in order to interact with digital information, found in the majority of modern consumer electronics, mainly computers, tablets and mobile phones [38].

### Procedures and design

After participants provided written informed consent, they were sent to a quiet isolated room to perform the tasks with only the researcher present. The computer monitor was positioned on a table in front of the participants. The chair was adjusted in accordance with the size and need of the individual, with a footrest available and for wheelchair users, their own wheelchair was used. After the necessary adjustments to perform the task, questionnaires and functional assessment scales were applied; the researcher provided verbal instructions and performed a demonstration of interfaces according to each task (motion tracking, finger motion control or touchscreen). Participants were instructed to use the dominant hand (i.e., the least affected side) for all interfaces used during the game.

To interact with the task, the individual was instructed to wave the hand in front of the sensor for motion tracking (Fig. 1a), wave the fingers above the sensor for Finger Motion Control (Fig. 1b) or touch the screen of the computer for Touchscreen interface (Fig. 1c). Figure 2 presents the task design used in this study.

### Data analysis

The dependent variable was the number of bubbles touched in each period. An ANOVA with between factors being Group (ALS, Control) and Interfaces (motion tracking, touchscreen, finger motion control), and attempts as within factor, with repeated measures on the last factor. For ‘attempts, separate comparisons were made in the acquisition phase (first acquisition attempt - FA; versus last acquisition attempt - LA), retention (LA versus retention attempt - R) and transfer (R versus first transfer attempted – T1 and R versus second transfer attempt - T2). Partial eta- squared (η^2^) was reported to measure effect size and interpreted as small (effect size > 0.01), medium (effect size > 0.06), or large (effect size > 0.14) [39]. Post-hoc comparisons were performed using Tukey’s Honest Significant Difference (HSD) test. Regression analysis considering improvement in movement time in the first and last attempt (⊗) was performed to determine which factors (age, gender, ALSFRS, ALSFSS, Daily Life Activities, Mobility, Swallowing, Communication, Emotional and ALSAQ-40/BR) influenced the degree of learning during practice for the ALS group. We considered findings to be significant at *p* < 0.05. The software package used was SPSS 20.0.

## Results

Both the ALS and Control groups were matched for age and gender, the values are the same for either group. A total of 10 individuals were allocated to each subgroup for the different interaction devices, with a mean age of 58 ± 11 years (3 women, 30% and 7 men, 70%) in the Motion Tracking group, mean age of 59 ± 9 and (3 women, 30% and 7 men, 70%) in the Touchscreen group and mean age of 61 ± 12 (5 women, 50% and 5 men, 70%) in the Finger Motion Control group. For the ALS group, Table 1 presents the characteristics of individuals, including duration of disease, ALSFRS-R, FSS and ALSAQ-40/BR rating scales, which were applied at the Neuromuscular Disease Research Sector of the Federal University of São Paulo. Clinical presentation of symptoms ranged from 13 to 212 months, and this was due to two patients with a diagnosis of 199 and 212 months which increased the mean and standard deviation, yet disease duration was not associated with more severe symptoms; All individuals with ALS were able to complete the task. The ALSFRS-R functionality scale indicated that 40% of individuals with ALS had fatigue.

**Table 1:** Characterization of individuals with ALS, attended at Neuromuscular Disease Research Sector at the Federal University of São Paulo, according to the duration of disease, age and rating scales

	Group with acquisition and retention on:			
*n*	*Motion Tracking*	*Touchscreen*	*Finger Motion Control*	p*
	*10*	*10*	*10*	
	*Mean (SD)*	*Mean (SD)*	*Mean (SD)*	
Disease duration (months)	74 (75)	35 (22)	29 (12)	0.077
ALSFRS-R	31.3 (7.48)	31.0 (5.56)	24.7 (7.04)	0.129
FSS	37 (18)	37 (17)	46 (12)	0.359
Mobility	67 (18)	62 (27)	84 (24)	0.097
DLAs	55 (23)	56 (27)	78 (30)	0.122
Swallowing;	31 (33)	28 (41)	57 (29)	0.137
Communication	37 (37)	23 (26)	56 (37)	0.099
Emotional	52 (27)	29.2 (15)	56 (29)	0.043*
ALSAQ-40/BR (Total)	229 (100)	199 (78)	332 (102)	0.010*

n: sample size; ALSFRS-R: Revised Amyotrophic Lateral Sclerosis Functional Rating Scale; FSS: Fatigue Severity Scale; ALSAQ-40/BR (Total): Amyotrophic Lateral Sclerosis Assessment Questionnaire in the Portuguese Language; DLAs: daily living activities. ANOVA (*p* < 0.05): *post-hoc test showed differences between the groups Motion Tracking and touchscreen and between touchscreen and Finger Motion Control

### Results in protocol phases

#### Acquisition

The results are represented in Fig. 3. Significant effects were found for Attempts [F_1, 51_ = 50.1, *p* < 0.001, ŋ^2^ = 0.50], Groups [F_1, 51_ = 21.3, *p* < 0.001, ŋ^2^ = 0.29] and Interfaces [F_2, 51_ = 9.3, *p* < 0.001, ŋ^2^ = 0.27]. This result suggests that both groups increased the number of bubbles reached from First Attempt (FA) (M = 62) to Last Attempt (LA) (M = 83) and that the ALS group showed worse performance (M = 62) compared to the CG (M = 83). A significant interaction between Group and Interfaces [F_2, 51_ = 6.8, *p* = 0.002, ŋ^2^ = 0.21] was found. The post hoc test showed that the worse performance occurred on the touch screen between the groups (*p* < 0.001; M = 107 and 65, respectively) and finger motion control (*p* = 0.009; M = 72 and 50, respectively) yet not on the motion tracking control device. In addition, participants from both groups who performed acquisition on touchscreen performed significantly better (M = 90) compared with motion tracking (*p* = 0.007; M = 70) and finger motion control (*p* < 0.001; M = 63), but there were no significant differences between motion tracking and finger motion control.

#### Retention

There was a significant effect for Interfaces [F_2, 53_ = 9.2, *p* < 0.001, ŋ^2^ = 0.26] which showed that touchscreen promoted better performance for all individuals (M = 99), compared to motion tracking (M = 77) and finger motion control (M = 79). There was a significant interaction between Attempts and Interfaces [F_2, 53_ = 3.2, *p* = 0.048, ŋ^2^ = 0.11]. The post hoc test showed that there was significant improvement in the amount of bubbles reached from the last acquisition block (M = 75) to the retention block (M = 83; *p* = 0.007) in the finger motion control. There was no difference between blocks on motion tracking and touchscreen for either group. The main effect for Groups [F_1, 54_ = 25.1, *p* < 0.001, ŋ^2^ = 0.32] and Interfaces [F_2, 54_ = 9.36, *p* < 0.001, ŋ^2^ = 0.26] remained present. There was also interaction between Groups and Interfaces [F_2, 54_ = 4.62, *p* = 0.014, ŋ^2^ = 0.15]. As in the acquisition phase, the results showed that there were significant differences between the CG and ALS groups on touchscreen (*p* < 0.001; M = 116 and 82, respectively) and finger motion control (*p* = 0.001; M = 94 and 64, respectively), but there was no significant difference in motion tracking (Fig. 3).

#### Transfer 1

Significant effects were found for Attempts [F_1, 54_ = 16.7, *p* < 0.001, ŋ^2^ = 0.24] and Groups [F_1, 54_ = 32.1, *p* < 0.001, ŋ^2^ = 0.37]. This result suggested that all individuals decreased the number of bubbles touched from R (M = 86) to T1 (M = 74) and additionally, the CG had a higher number of bubbles touched (M = 92) compared to the ALS group (M = 68). Interaction between Attempts and Interfaces [F_2, 54_ = 34.5, *p* < 0.001, ŋ^2^ = 0.56] and Groups and Interfaces [F_2, 54_ = 3.7, *p* = 0.031, ŋ^2^ = 0.12] were found. The post-hoc test showed that the group that carried out the Acquisition/Retention phase in motion tracking (Retention M = 77) improved performance in transfer with touchscreen (M = 93; *p* = 0.003), but when practice was made with touchscreen (Retention M = 98) performance deteriorated in the transfer to motion tracking (M = 56; *p* < 0.001). There were no significant differences between acquisition/retention in finger motion control and transfer to motion tracking. In addition, there were differences between the groups in touchscreen (*p* = 0.001; M = 91 and 64, respectively) and finger motion control (*p* < 0.001; M = 97 and 60, respectively), but not in motion tracking (Fig. 3).

#### Transfer 2

Significant effects were found for Attempts [F_1, 54_ = 19.5, *p* < 0.001, ŋ^2^ = 0.27] and Groups [F_1, 54_ = 14.0, *p* < 0.001, ŋ^2^ = 0 21]. These results suggested that individuals decreased the number of bubbles reached in R (M = 86) to T2 (M = 72). Moreover, the CG had a higher number of bubbles touched (M = 88) compared to ALS (F = 70). The results showed interaction between Attempts and Interfaces [F_2, 54_ = 12.8, *p* < 0.001, ŋ^2^ = 0.32]. The post hoc test found that the group undergoing the stages of Acquisition/Retention on touchscreen (Retention M = 98) had worse performance on transfer to finger motion control (*p* < 0.001; M = 62), but there were no significant differences between Acquisition/Retention in motion tracking and transfer for finger motion control, or between Acquisition/Retention in finger motion control and transfer to touchscreen (Fig. 3).

#### The effect of practice on the transfer phase

In order to verify if a transfer effect occurred or if it was only a characteristic of the task (easier than the interface of practice), t-tests were performed to compare the performance on transfer and the first attempt (FA) of the group that started in the same interface (e.g. transfer T1 on touchscreen of Group B versus FA on touchscreen of Group A, transfer T1 on motion tracking of Group C versus FA on motion tracking of Group B, transfer T1 on finger motion control of Group A versus FA on finger motion control of Group C, see Fig. 3). The results showed that only for the ALS group that had performed the acquisition phase on motion tracking [with T1 phase on touchscreen (M = 87)] presented statistical difference from the ALS group that performed the first acquisition attempt on the touchscreen (M = 54, *p* = 0.035). This implies that the practice on motion tracking (without physical contact) promoted better performance when transferring to touchscreen (with physical contact) for individuals with ALS, in other words they performed better on the task with physical contact after practice on motion tracking than when they started with touchscreen (Fig. 3).

#### Linear regression

None of the factors were found to influence the degree of learning during practice of the task, which indicates that performance was not affected by differences in age or gender, or the scores in ALSFRS, ALSFSS, DLAs, Mobility, Swallowing, Communication, Emotional or ALSAQ-40/BR.

## Discussion

The purpose of this study was to investigate the use of different interaction devices (used in daily life) that may improve performance in individuals with ALS. Moreover, our interest was to determine if a more abstract device (without physical contact) could provide better performance than a device with physical contact in a VR task. Our results showed better performance on the physical contact device (touchscreen) for both the ALS and CG, offering greater ability for those individuals to perform tasks and retain skills during the phases of acquisition and retention. This result was contrary to the initial hypothesis in which we expected that finger motion control which is characterized by being a device without physical contact that does not require accurate touching nor a wide range of movement, should be more functional than the touchscreen which needs more precise control of movement. Our findings are important as they confirm the utility of touchscreen systems to promote functionality on daily tasks using a computer in individuals with ALS.

Although there was better performance on the touchscreen for both the ALS and CG, all participants were found to have improvements in performance with practice regardless of the interaction device. This was expected as even with the characteristic of progressive neuromuscular changes and possible cognitive impairments that interfere with performance [40], individuals with ALS have been shown to retain the capacity to learn and our results showed that individuals with ALS are able to make compensatory movements to accomplish the task and improve performance, even when using different VR interaction devices. Corroborating this finding, Lancioni et al. [18] (2011), stated that individuals with ALS have the capacity to improve performance and learn new tasks using computer and coupled devices. Also, the authors state that these kinds of tasks promote engagement on the activities, which is an important characteristic due to the fact that the lack of engagement in therapy is the biggest barrier for rehabilitation of people with disabilities [41].

Regarding the comparison between groups, the CG showed better performance than the ALS group in most of the devices in all phases of the study, except when using motion tracking on the acquisition and retention phases. We can only speculate that, the difficulty to organize movement during motion tracking practice was the same for both groups, even with the motor and possible cognitive impairments that characterizes ALS. Therefore, this type of device may allow equal performance for individuals with ALS compared to healthy controls to enjoy social activities with families and friends.

Besides the acquisition and retention phases, the ability to transfer skills is an important finding as it shows that there was motor learning (short-term), and not just improvements in performance [42] (i.e. if they have the ability to transfer, the learning can be inferred). Thus, for the transfer phase with change of device, we mainly observed that the groups that made acquisition with touchscreen failed to transfer the task to the devices without physical contact. However, participants in the ALS group who in the acquisition phase performed the task without physical contact performed better when transferred to the touchscreen. This is shown by the performance in the touchscreen after using the motion tracking device (M = 87) which showed improvement compared to the ALS group who began on the touchscreen (M = 54). This finding may be considered to encourage utilization of technologies in clinical practice and therefore has great novelty and potential impact. Despite the difficulty of using a device without physical contact, the exposure to this virtual environment enabled better performance when the individuals underwent transfer to a more realistic environment (represented by the touchscreen). This concept added to the possibility of VR environments (mainly using no contact device) being considered more motivating to use, enabling control of quality, intensity, duration and frequency of exposure of the individual to the task, which resulted in benefits when transferring to other devices [43].

These findings are in line with Monteiro et al. [27] and Massetti et al. [13] who carried out comparisons between real and virtual environments in individuals with neuromuscular disease and found that a direct interaction with the environment through physical contact provided a richer set of information to guide movement and better performance. According to Massetti et al. [13] despite the playful features, ease of access and the power of fascination that VR can create in individuals, its use should be carefully considered as often the task features make it much more difficult and complex than its real representation.

Moreover, some complementary explanations may contribute to the justification for the difficulty found in using a no contact device. Lack of familiarity with the systems with no physical contact may have hampered performance in these devices in comparison to touchscreen devices which are widely used in everyday life. This would be in agreement with Bulmaro et al. [44], who found that when using a VR task with no physical contact, individuals needed additional training time and a prolonged period of practice.

A second influence may be failures of the calibration of the device itself [43]. We observed during the data collection that both the motion tracking and finger motion devices showed some slight technical delays (freezing images or delays in response to the movement) while performing the task, which were difficult to identify as this was due to shortcomings in the developed software and/or were due to the less than optimal sensitivity of the devices. Iosa et al. [43] found that the finger motion control is an important tool for rehabilitation but mentions a limitations in tracking finger movements during activities when the hands gets too close to the motion sensor. We found similar issues in the current study.

The third influence may be the absence of tactile feedback when using a no contact device. Tactile feedback may include touch sensation, temperature, and surface friction and can increase the sensitivity of stimuli present, providing an efficient communication channel [13]. According to Monteiro et al. [27] a task that involves a direct interaction with the environment including physical contact generates a richer pool of information for guiding movement compared to a more abstract task, in a virtual environment. Hence, the two tasks with no physical contact depend on different information–movement couplings and it is not unlikely therefore that this environment may elicit different spatio-temporal organization of the movement than natural environments, especially among participants with movement disorders. However, it is also known that when the practice is in some way more difficult and requires more spatio-temporal organization, there is a better transfer of learning then when the task offers an easier practice [45], as confirmed by our results. Although using the touchscreen was easier, the devices that did not required physical contact promoted greater transfer of the task. Thus, we can speculate that touchscreen devices can be used to promote functionality on daily tasks using a computer, however, if the aim of the rehabilitation team is to improve this functionality, it is important to consider using devices with no physical contact.

To mitigate any bias around motor differences between ALS groups that could influence the results, three assessment scales were used to verify disease stage, fatigue and quality of life of individuals with ALS across groups. We found that there were no significant differences with homogeneity in disability and motor skills between groups. The only exception was in the “*Emotional”* domain of the ALSAQ-40/BR quality of life scale in the group that started with the touchscreen task.

Despite the possible sensitivity limits of the virtual devices, varying characteristics of participants, number of participants and low repetitions for adjustments to task, clear strong effects were found. This study suggests that for the first time a touchscreen system could help to improve performance in individuals with ALS, in addition to the potential for using technologies to improve functionality in this group. It is important to emphasize that a game was used to test performance and that we anticipate transfer to other tasks, but this work needs replicating with other technological devices including communication and socialization systems which have been found to promote self- determination, increase patients’ quality of life and reduce caregiver burden [46]. Providing the best device for an individual to perform practical tasks is extremely important, considering health service resources and the impact on participation in meaningful activities of this condition. In this group of individuals with ALS, it became clear that for them the touchscreen was the most functional device. Finally, equal performance was observed using the motion tracking between the CG and ALS suggesting this device may be a good system to allow equal competition if used for gaming and for leisure.

### Limitations and future studies

Although we found interesting results, we can point out some limitations: (1) we can consider the lack of familiarity with the devices without physical contact as a limitation, but this is a factor that can be controlled with training and practice and should be investigated in futures studies; (2) Delays and technical issues of the devices with no physical contact could impact the results, however we believe this was out of the control of the researcher (3) A VR task was used to assess the motor performance that could be extrapolated for communication (an important function for ALS individuals), however future studies should focus on tasks specific for augmentative and alternative communication (AAC) devices; (4) In order to guarantee the homogeneity of the ALS group, we did not include individuals with Bulbar-onset ALS, familial ALS, Progressive muscular atrophy (PMA) and Primary lateral sclerosis (PLS) as it would be difficult to measure the influence of the type of ALS on the results; however this means that the results from the current study may not be translated to other types of ALS; (5) our results cannot be extrapolated to later stages of the disease, with high-grade deficits of the upper extremities or locked-in syndrome, in which Brain-Computer Interfaces or eye tracker are more suitable technologies.

## Conclusion

The study demonstrated that individuals with ALS were better able to use a touchscreen device to perform tasks on a computer than devices with no physical contact. Moreover, we found a clinically important result that when using a motion tracking device (without physical contact) individuals with ALS could transfer performance to a touchscreen. Our findings are important as they suggest that individuals with ALS with moderate functional impairment may find touchscreen devices easier for improving and retaining motor performance, and therefore may have application to daily life tasks using a computer system. In addition, when considering rehabilitation to improve motor abilities of individuals with ALS on touchscreen devices, it is important to consider using motion track devices for practice of the task.

## Abbreviations

ALS: Amyotrophic Lateral Sclerosis
ALSAQ-40/BR: Amyotrophic Lateral Sclerosis Assessment Questionnaire in the Portuguese Language
ALSFRS – R: Revised Amyotrophic Lateral Sclerosis Functional Rating Scale
FSS: Fatigue Severity Scale
PBP: Progressive bulbar paralysis
PLS: Primary lateral sclerosis
PMA: Progressive muscular atrophy
SIDNM: Neuromuscular Disease Research Sector
VR: Virtual Reality

## References

[1] Mulder DW (1982). Clinical limits of amyotrophic lateral sclerosis. Adv Neurol.

[2] Couratier P, Corcia P, Lautrette G, Nicol M, Preux PM, Marin B (2016). Epidemiology of amyotrophic lateral sclerosis: a review of literature. Rev Neurol (Paris).

[3] Logroscino G, Traynor BJ, Hardiman O, Chio A, Mitchell D, Swingler RJ (2010). Incidence of amyotrophic lateral sclerosis in Europe. J Neurol Neurosurg Psychiatry.

[4] Handy CR, Krudy C, Boulis N, Federici T (2011). Pain in amyotrophic lateral sclerosis: a neglected aspect of disease. Neurol Res Int.

[5] Eisen A, Kuwabara S (2012). The split hand syndrome in amyotrophic lateral sclerosis. J Neurol Neurosurg Psychiatry.

[6] Menon P, Kiernan MC, Vucic S (2014). ALS pathophysiology: insights from the split-hand phenomenon. Clin Neurophysiol.

[7] Cook AM, Polgar JM. Assistive technologies-E-book: principles and practice: Elsevier Health Sciences; 2014.

[8] Londral A, Pinto A, Pinto S, Azevedo L, De Carvalho M (2015). Quality of life in amyotrophic lateral sclerosis patients and caregivers: impact of assistive communication from early stages. Muscle Nerve.

[9] Funke A, Spittel S, Grehl T, Grosskreutz J, Kettemann D, Petri S (2018). Provision of assistive technology devices among people with ALS in Germany: a platform-case management approach. Amyotroph Lateral Scler Frontotemporal Degener.

[10] Nam CS, Woo J, Bahn S (2012). Severe motor disability affects functional cortical integration in the context of brain–computer interface (BCI) use. Ergonomics.

[11] Crocetta TB, de Araújo LV, Guarnieri R, Massetti T, Ferreira FHIB, de Abreu LC (2018). Virtual reality software package for implementing motor learning and rehabilitation experiments. Virtual Reality.

[12] Henderson A, Korner-Bitensky N, Levin M (2007). Virtual reality in stroke rehabilitation: a systematic review of its effectiveness for upper limb motor recovery. Top Stroke Rehabil.

[13] Massetti T, Fávero FM, LDCd M, Alvarez MPB, Crocetta TB, Guarnieri R (2018). Achievement of virtual and real objects using a short-term motor learning protocol in people with duchenne muscular dystrophy: a crossover randomized controlled trial. Games health J.

[14] Saposnik G, Cohen LG, Mamdani M, Pooyania S, Ploughman M, Cheung D (2016). Efficacy and safety of non-immersive virtual reality exercising in stroke rehabilitation (EVREST): a randomised, multicentre, single-blind, controlled trial. Lancet Neurol.

[15] da Silva TD, de Mello Monteiro CB, Corrêa AGD, Alonso AC, Greve JMDA. REALIDADE VIRTUAL NA PARALISIA CEREBRAL Definição, tipos e possibilidades de intervenção. PARALISIA CEREBRAL. 2015;1:249.

[16] Silvoni S, Cavinato M, Volpato C, Ruf CA, Birbaumer N, Piccione F (2013). Amyotrophic lateral sclerosis progression and stability of brain-computer interface communication. Amyotroph Lateral Scler Frontotemporal Degener.

[17] Thompson DE, Gruis KL, Huggins JE (2014). A plug-and-play brain-computer interface to operate commercial assistive technology. Disabil Rehabil Assist Technol.

[18] Lancioni GE, Singh NN, O’Reilly MF, Green VA, Ferlisi G, Ferrarese G (2013). A man with amyotrophic lateral sclerosis uses a mouth pressure microswitch to operate a text messaging system with a word prediction function. Dev Neurorehabil.

[19] Caligari M, Godi M, Guglielmetti S, Franchignoni F, Nardone A (2013). Eye tracking communication devices in amyotrophic lateral sclerosis: impact on disability and quality of life. Amyotroph Lateral Scler Frontotemporal Degener.

[20] Linse K, Rüger W, Joos M, Schmitz-Peiffer H, Storch A, Hermann A (2018). Usability of eyetracking computer systems and impact on psychological wellbeing in patients with advanced amyotrophic lateral sclerosis. Amyotroph Lateral Scler Frontotemporal Degener.

[21] Linse K, Rüger W, Joos M, Schmitz-Peiffer H, Storch A, Hermann A (2017). Eye-tracking–based assessment suggests preserved well-being in locked-in patients. Ann Neurol.

[22] Cohen AR, Lohani S, Manjila S, Natsupakpong S, Brown N, Cavusoglu MC (2013). Virtual reality simulation: basic concepts and use in endoscopic neurosurgery training. Childs Nerv Syst.

[23] Huber M, Rabin B, Docan C, Burdea GC, AbdelBaky M, Golomb MR (2010). Feasibility of modified remotely monitored in-home gaming technology for improving hand function in adolescents with cerebral palsy. IEEE Trans Inf Technol Biomed.

[24] Hurkmans HL, Van den Berg-Emons RJ, Stam HJ (2010). Energy expenditure in adults with cerebral palsy playing Wii sports. Arch Phys Med Rehabil.

[25] Vissers M, Van den Berg-Emons R, Sluis T, Bergen M, Stam H, Bussmann H (2008). Barriers to and facilitators of everyday physical activity in persons with a spinal cord injury after discharge from the rehabilitation Centre. J Rehabil Med.

[26] Wilson PN, Foreman N, Stanton D (1997). Virtual reality, disability and rehabilitation. Disabil Rehabil.

[27] de Mello Monteiro CB, Massetti T, da Silva TD, van der Kamp J, de Abreu LC, Leone C (2014). Transfer of motor learning from virtual to natural environments in individuals with cerebral palsy. Res Dev Disabil.

[28] Faria DC, Fávero FM, Fontes SV, Quadros AAJ, Oliveira ASB (2008). Perfil clinico de pacientes com doença do neuronio motor no ambulatorio da Unifesp. Rev Neurocienci.

[29] Ross MA, Miller R, Berchert L, Parry G, Barohn R, Armon C (1998). Toward earlier diagnosis of amyotrophic lateral sclerosis revised criteria. Neurology.

[30] Guedes K, Pereira C, Pavan K, Valério BCO (2010). Cross-cultural adaptation and validation of als functional rating scale-revised in Portuguese language. Arq Neuropsiquiatr.

[31] Krupp LB, LaRocca NG, Muir-Nash J, Steinberg AD (1989). The fatigue severity scale: application to patients with multiple sclerosis and systemic lupus erythematosus. Arch Neurol.

[32] Pavan K, Marangoni BE, Zinezzi MO, Schmidt KB, Oliveira BC, Buainain RP (2010). Validation of the amyotrophic lateral sclerosis assessment questionnaire (ALSAQ-40) scale in the portuguese language. Arq Neuropsiquiatr.

[33] Jenkinson C, Levvy G, Fitzpatrick R, Garratt A (2000). The amyotrophic lateral sclerosis assessment questionnaire (ALSAQ-40): tests of data quality, score reliability and response rate in a survey of patients. J Neurol Sci.

[34] DiFilippo NM, Jouaneh MK (2015). Characterization of different Microsoft Kinect sensor models. IEEE Sensors J.

[35] Zhao K, Luximon A, Balasankar G, Chan C (2014). A new representational method of human foot anatomical landmark and its application in foot posture data acquisition. Adv Appl Digital Human Model.

[36] Fujimura M, Sato S, Higashi T, Oguri K, editors. Study of mirror box therapy support system by leap motion. Consumer Electronics-Taiwan (ICCE-TW), 2015 IEEE International Conference on; 2015: IEEE.

[37] Marin G, Dominio F, Zanuttigh P, editors. Hand gesture recognition with leap motion and kinect devices. Image Processing (ICIP), 2014 IEEE International Conference on; 2014: IEEE.

[38] Hou Z, Zhang Y, Yang Y, editors. Enhancing touch screen games through a cable-driven force feedback device. Virtual Reality and Visualization (ICVRV), 2012 International Conference on; 2012: IEEE.

[39] Cohen J (1988). Statistical power analysis for the behavioral sciences.

[40] Swinnen B, Robberecht W (2014). The phenotypic variability of amyotrophic lateral sclerosis. Nat Rev Neurol.

[41] Lohse K, Shirzad N, Verster A, Hodges N, Van der Loos HM (2013). Video games and rehabilitation: using design principles to enhance engagement in physical therapy. J Neurol Phys Ther.

[42] de Mello Monteiro CB, da Silva TD, de Abreu LC, Fregni F, de Araujo LV, Ferreira FHIB (2017). Short-term motor learning through non-immersive virtual reality task in individuals with Down syndrome. BMC Neurol.

[43] Iosa M, Morone G, Fusco A, Castagnoli M, Fusco FR, Pratesi L (2015). Leap motion controlled videogame-based therapy for rehabilitation of elderly patients with subacute stroke: a feasibility pilot study. Top Stroke Rehabil.

[44] Bulmaro A, Van der Loos H. Biofeedback vs. game scores for reducing trunk compensation after stroke: a randomized crossover trial (vol 25, pg 96, 2017). Top Stroke Rehabil 2018;25(3):239-. 10.1080/10749357.2017.1394633 29078743

[45] Lin C-HJ, Yang H-C, Knowlton BJ, Wu AD, Iacoboni M, Ye Y-L (2018). Contextual interference enhances motor learning through increased resting brain connectivity during memory consolidation. NeuroImage.

[46] Linse K, Aust E, Joos M, Hermann A (2018). Communication matters–pitfalls and promise of hightech communication devices in palliative care of severely physically disabled patients with amyotrophic lateral sclerosis. Front Neurol.

